# Th1/Th2 Imbalance in Peripheral Blood Echoes Microglia State Dynamics in CNS During TLE Progression

**DOI:** 10.1002/advs.202405346

**Published:** 2024-08-13

**Authors:** Jing Wang, Yuanxia Wu, Jing Chen, Qiong Zhang, Yunyi Liu, Hongyu Long, Jianhua Yu, Qian Wu, Li Feng

**Affiliations:** ^1^ Department of Neurology Xiangya Hospital Central South University Changsha Hunan 410008 China; ^2^ National Clinical Research Center for Geriatric Disorders Xiangya Hospital Central South University Changsha Hunan 410008 China; ^3^ Department of Neurology Guizhou Provincial People's Hospital Guiyang Guizhou 550002 China; ^4^ State Key Laboratory of Oral Diseases & National Center for Stomatology & National Clinical Research Center for Oral Diseases & Department of Operative Dentistry and Endodontics West China Hospital of Stomatology Sichuan University Chengdu Sichuan 610041 China; ^5^ Department of Immuno‐Oncology City of Hope Los Angeles CA 91010 USA; ^6^ Department of Neurology First Affiliated Hospital Kunming Medical University Kunming Yunnan 650032 China

**Keywords:** central inflammation, Notch1, systemic inflammation, temporal lobe epilepsy

## Abstract

Central and systemic inflammation play pivotal roles in epileptogenesis and proepileptogenesis in temporal lobe epilepsy (TLE). The interplay between peripheral CD4^+^ T cells and central microglia orchestrates the “systemic‐central” immune response in TLE. However, the precise molecular mechanisms linking central and systemic inflammation in TLE remain unknown. This preliminary findings revealed an imbalance in Th1/Th2 subsets in the periphery，accompanied by related cytokines release in TLE patients. they proposed that this peripheral Th1/Th2 imbalance may influence central inflammation by mediating microglial state dynamics within epileptic foci and distant brain regions. In Li‐pilocarpine‐induced TLE rats, a peripheral Th1/Th2 imbalance and observed corresponding central and systemic responses is confirmed. Notably, CD4^+^ T cells infiltrated through the compromised blood‐brain barrierand are spatially close to microglia around epileptic foci. Intravenous depletion and reinfusion of CD4^+^ T cells modulated microglia state dynamics and altered neuroinflammatory cytokines secretion. Moreover, mRNA sequencing of the human hippocampus identified Notch1 as a key regulator of Th1/Th2 differentiation, CD4^+^ T cell recruitment to brain infiltration sites, and the regulation of microglial responses, seizure frequency, and cognition. This study underscores the significance of Th1/Th2 imbalance in modulating the “systemic‐central” response in TLE, highlighting Notch1 as a potential therapeutic target.

## Graphical abstract

This study identifies a significant association between peripheral Th1/Th2 imbalance and central microglia state dynamics during temporal lobe epilepsy (TLE) progression. It underscores the complex interaction between peripheral CD4^+^ T cells and central microglia in orchestrating the ‘systemic‐central’ immune response in TLE, with Notch 1 emerging as a potential therapeutic target to mitigate central inflammation.

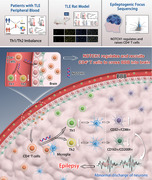

## 1 Introduction

Central inflammation is an inflammatory process occurring within the central nervous system (CNS) that involves the participation of immune cells and various signaling molecules. In the context of temporal lobe epilepsy (TLE), numerous studies have consistently reported evidence supporting the presence of central inflammatory mechanisms.^[^ ^1^, ^2^, ^3^ ^]^ Across specimens resected from the epileptic foci of patients and animal models, multiple inflammation‐related pathways in the CNS, including the TORC1 pathway, IL‐1 receptor 1‐Toll‐like receptor 4 axis, arachidonic acid prostanoid cascade, and oxidative stress response, have been shown to be activated.^[^ ^4^ ^]^ Advanced imaging techniques, such as positron emission tomography (PET) for evaluating translocator protein expression or proton magnetic resonance spectroscopy, have notably depicted heightened activation of integral components of central inflammation within epileptogenic regions in patients with mesial TLE.^[^ ^5^, ^6^, ^7^ ^]^ Pathophysiologically, the consequences of central inflammation include alterations in neuronal excitability, synaptic function, and network dynamics, which collectively influence seizure initiation and propagation. Seizures themselves can serve as triggers for inflammatory responses, establishing a reciprocal relationship wherein inflammation contributes to seizures ^[^ ^8^ ^]^ and, in turn, exacerbates inflammation. Moreover, inflammation in the CNS is believed to contribute to both ictogenic and epileptogenic processes, marked by the transformation of normal brain tissue into a state that is more susceptible to seizures.

However, central inflammation is not the sole contributor to TLE, peripheral inflammation also plays a significant role in its progression. Elevated levels of various inflammatory markers, such as C‐reactive protein and interleukin‐6 (IL‐6), IL‐1β, and tumor necrosis factor‐α (TNF‐α) have been detected in the peripheral blood of TLE patients and animal models.^[^ ^9^, ^10^ ^]^ These findings underscore the systemic nature of inflammation in TLE and provide direct evidence of the involvement of peripheral immune responses in this disorder. Blood‐brain barrier (BBB) integrity is compromised in epilepsy, including TLE, which potentially facilitates the infiltration of immune cells, notably CD4^+^ T cells, into the brain parenchyma.^[^ ^4^ ^]^ Notably, CD4^+^ T cells have been implicated in epilepsy‐related peripheral inflammation in TLE patients and rodent models.^[^ ^10^, ^11^, ^12^ ^]^ Inflammatory conditions elsewhere in the body can influence the CNS and potentially contribute to neuronal hyperexcitability. For example, in Theiler's murine encephalomyelitis virus‐induced encephalitis mice, the infiltration of peripheral macrophages into the brain is pivotal for the induction of seizures accompanied by increased IL‐6 production. The inhibition of this infiltration is effective for treating seizures. This bidirectional communication between the CNS and peripheral immune system emphasizes the complexity of TLE. Understanding the interplay between central and peripheral inflammation is crucial for developing targeted therapeutic strategies to address both the local and systemic aspects of the inflammatory response in TLE.

The intricate interplay between microglia and CD4^+^ T cells plays a pivotal role in the immune response and contributes to the central inflammatory response observed in TLE. Microglia, the resident immune cells of the CNS, and CD4^+^ T cells of the adaptive immune system engage in dynamic interactions. Anatomically, their collaboration relies on epilepsy‐induced BBB compromise, which allows the infiltration of peripheral CD4^+^ T cells into the CNS.^[^ ^13^, ^14^ ^]^ Biochemically, various signaling molecules orchestrate the cooperation between these immune cells. Reactive microglia release cytokines and chemokines that influence local immune responses and attract CD4^+^ T cells to the site of inflammation.^[^ ^15^ ^]^ Conversely, CD4^+^ T cells release cytokines, further affecting microglia and prompting their polarization into pro‐inflammatory (M1) or anti‐inflammatory (M2) types responding to diseases.^[^ ^16^ ^]^ Additionally, microglia act as antigen‐presenting cells, present antigens to CD4^+^ T cells, and stimulate immune responses in the CNS.^[^ ^17^ ^]^ This complex interaction, along with the cytokine milieu, modulates the inflammatory environment, affects neuronal function, and potentially contributes to the development and progression of epilepsy. However, studies have shown the dynamic nature of the “systemic‐central” immune response in TLE, with immune cell subtypes and cytokines exhibiting both protective and detrimental roles. Excessive or dysregulated immune responses may contribute to neuronal damage and worsen seizure activity, whereas certain immune processes may be neuroprotective. Understanding the dynamic interplay between microglia and CD4^+^ T cells in TLE is crucial for deciphering the mechanisms underlying central inflammation in epilepsy, exploring therapeutic avenues to modulate inflammatory responses, and mitigating the effects of seizures.

In the present study, we identified an imbalance in the Th1/Th2 subsets of CD4^+^ T cells in the peripheral blood of patients with TLE. Concurrently, this imbalance was accompanied by the release of inflammatory cytokines in the plasma and neuronal injury in the CNS. Therefore, we hypothesized that this peripheral imbalance echoes microglia state dynamics in epileptogenic lesions or distant brain regions, influencing epilepsy progression. To test this hypothesis, we established a lithium (Li)‐pilocarpine rat model of epilepsy. Our objectives were to confirm the Th1/Th2 imbalance and related increase in cytokines in blood and brain tissues and to detect the localization of CD4^+^ T cells and microglia in the brain through immunofluorescence. Through the depletion and reinfusion of CD4^+^ T cells via intravenous injection, we aimed to investigate whether peripheral intervention with CD4^+^ T cells could modulate central microglia state dynamics and influence the secretion of inflammatory cytokines (IFN‐γ and IL‐4) in TLE. Notch1, identified through mRNA sequencing of human brain epileptogenic lesion tissue, regulates Th1/Th2 differentiation and recruitment of CD4^+^ T cells to brain infiltration sites.^[^ ^18^ ^]^ This study explored the feasibility of Notch1 inhibition as a therapeutic strategy for the treatment of epilepsy.

## 2 Results

### 2.1 Clinical and Demographic Characteristics

We enrolled 27 patients with TLE and 27 age‐ and sex‐matched healthy controls (HC). The clinical and demographic characteristics of the patients are summarized in **Table** **1**.

**Table 1:** Demographics of patients with TLE and healthy controls.

		TLE	HC	Significance
Age		32.5±10.3	32.3±10.0	0.8940
Sex	Female, n (% of total)	13(48%)	13(48%)	>0.9999
	Male, n (% of total)	14(52%)	14(52%)	
Onset age		24.5±9.2	‐	‐
Duration of seizures (years)		8±7.2	‐	‐
Seizure frequency (times/year)		8.2±6.3	‐	‐
Number of antiepileptic drugs		1.8±0.86	‐	‐

TLE, temporal lobe epilepsy; HC, healthy controls; Values are presented as the mean±SD.

### 2.2 Th1/Th2 Ratio was Increased and Correlated with Elevated Neuronal Injury Biomarker sNfL in the Peripheral Blood of Patients with TLE

**Figure 1:**
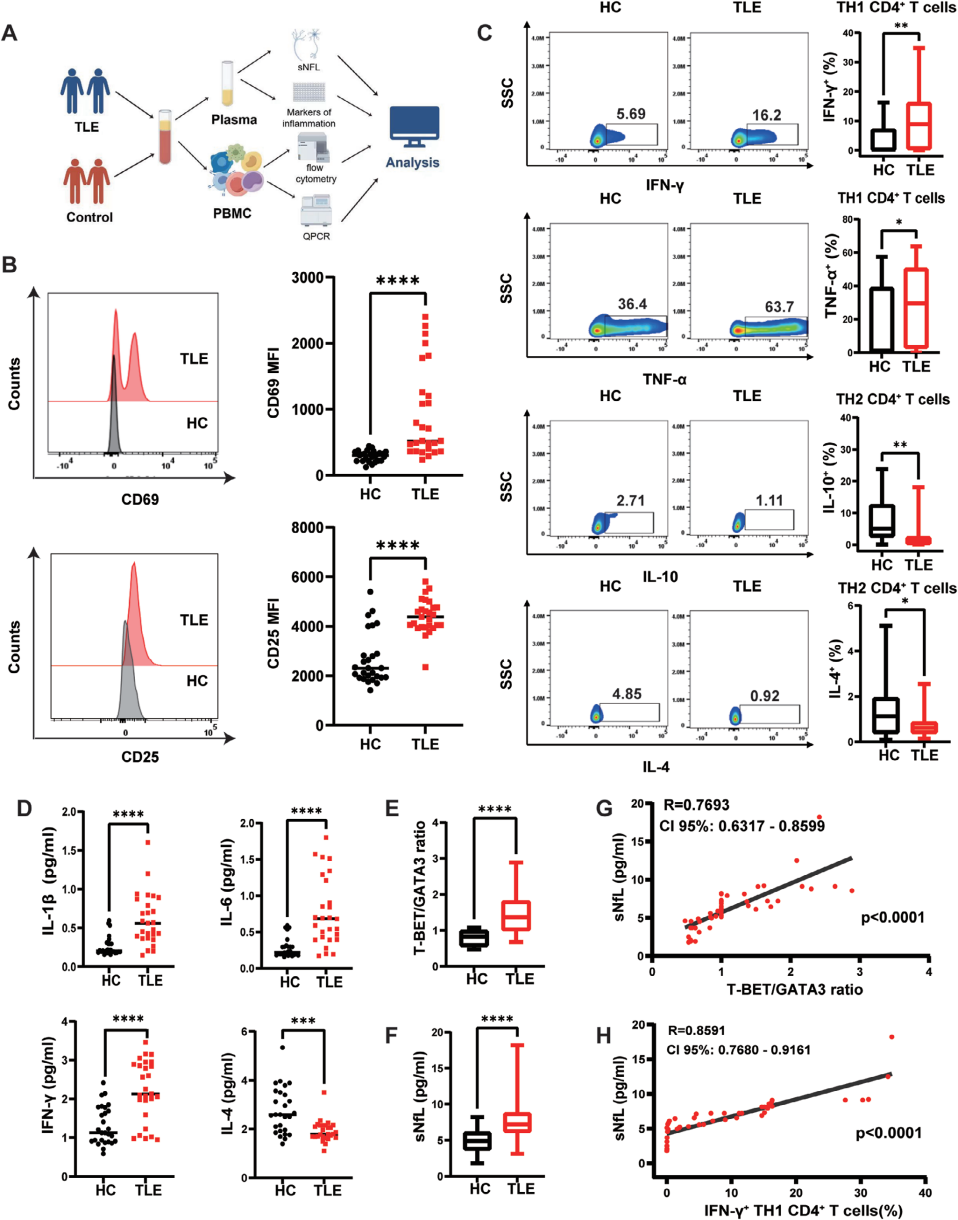
CD4^+^ T cells were activated and Th1/Th2 balance was disturbed in the peripheral blood from patients with TLE and was correlated with the concentration of sNfL. A) Flow chart of experimental scheme. B) Representative MFI curves and expression of activation markers (CD25, CD69) on the surface of CD4^+^ T cells from patients with TLE compared to those of healthy controls (HC). Quantification data are presented as mean MFI. C) Th1‐associated cytokine (IFN‐γ, TNF‐α) and Th2‐related intracellular cytokine (IL‐4, IL‐10) levels in CD4^+^ T cells determined by flow cytometry. D) Circulating levels of pro‐inflammatory (IL‐6, IFN‐γ, and IL‐1β) and anti‐inflammatory (IL‐4) cytokines in the serum of patients with TLE and HC determined by ELISA. E) The mRNA expression of transcription factor T‐bet and GATA3 were quantified using real‐time PCR. The T‐bet/GATA3 ratio was calculated. F) Expression of serum neurofilament light (sNfL) in patients with TLE compared to HC. Correlation (Pearson) of sNfL levels with T‐bet/GATA3 ratio G) and the proportion of IFN‐γ^+^ Th1 CD4^+^ T cells H). Patients with TLE, n = 27; HC, n = 27. Data are presented as mean ± standard deviation. *P<0.05, **P<0.01, ***P<0.001, ****P<0.0001.

Accumulating evidence suggests that peripheral immune cells infiltrate the CNS and contribute to epilepsy development.^[^ ^19^, ^20^ ^]^ A higher frequency of activated CD4^+^ T cells has been found in the peripheral blood of patients with epilepsy. Markers upregulated by activated CD4^+^ T cells allow them to interact with the BBB endothelium, allowing them to transmigrate across the BBB.^[^ ^14^, ^21^, ^22^ ^]^ To identify whether circulating CD4^+^ T cells were activated in patients with TLE, we extracted CD4^+^ T cells from their peripheral blood and performed flow cytometry analysis. The experimental procedure is illustrated in **Figure** **1**A. Compared with HC, the median fluorescence intensity (MFI) of markers associated with CD4^+^ T cell activation (HLA‐DR, CD69, and CD25) was significantly increased (Figure 1B; Figure S2A, Supporting Information). Activated Th1 CD4^+^ T cells express proinflammatory cytokines, whereas activated Th2 CD4^+^ T cells express anti‐inflammatory cytokines. To determine whether peripheral blood CD4^+^ T cells from patients with TLE were skewed toward a pro‐ or anti‐inflammatory phenotype, we detected cytokine production by flow cytometry after brief activation of isolated CD4^+^ T cells. Expression of Th1‐associated proinflammatory cytokines interferon (IFN)‐γ and tumor necrosis factor (TNF)‐α by CD4^+^ T cells were elevated in patients with TLE compared to HC. In contrast, the expression of Th2‐related anti‐inflammatory cytokines interleukin IL−4 and IL‐10 by CD4^+^ T cells were decreased (Figure 1C). Further, we measured peripheral cytokines implicated in T cell polarization by ELISA and observed IL‐1β, IL‐6, and IFN‐γ levels in serum from patients with TLE were increased compared to those of HC. In contrast, IL‐4 was detectable in a lower proportion of patients with TLE (Figure 1D). In addition, we found that the T‐bet (transcription factor for Th1 cell differentiation)/GATA3 (Th2 lineage‐committed transcription factor) ratio was increased (Figure 1E). Because pro‐inflammatory Th1 CD4^+^ T cells can exert neurotoxicity, we measured the serum levels of neurofilament light chain (sNfL), a biomarker of neuronal damage, and found that it was elevated in patients with TLE (Figure 1F). Furthermore, we found that the proportions of Th1 CD4^+^ T cells and Th2 CD4^+^ T cells, as well as the T‐bet/GATA3 ratio, were correlated with the levels of sNfL (Figure 1G,H; Figure S2B,C, Supporting Information).

**Figure 2:**
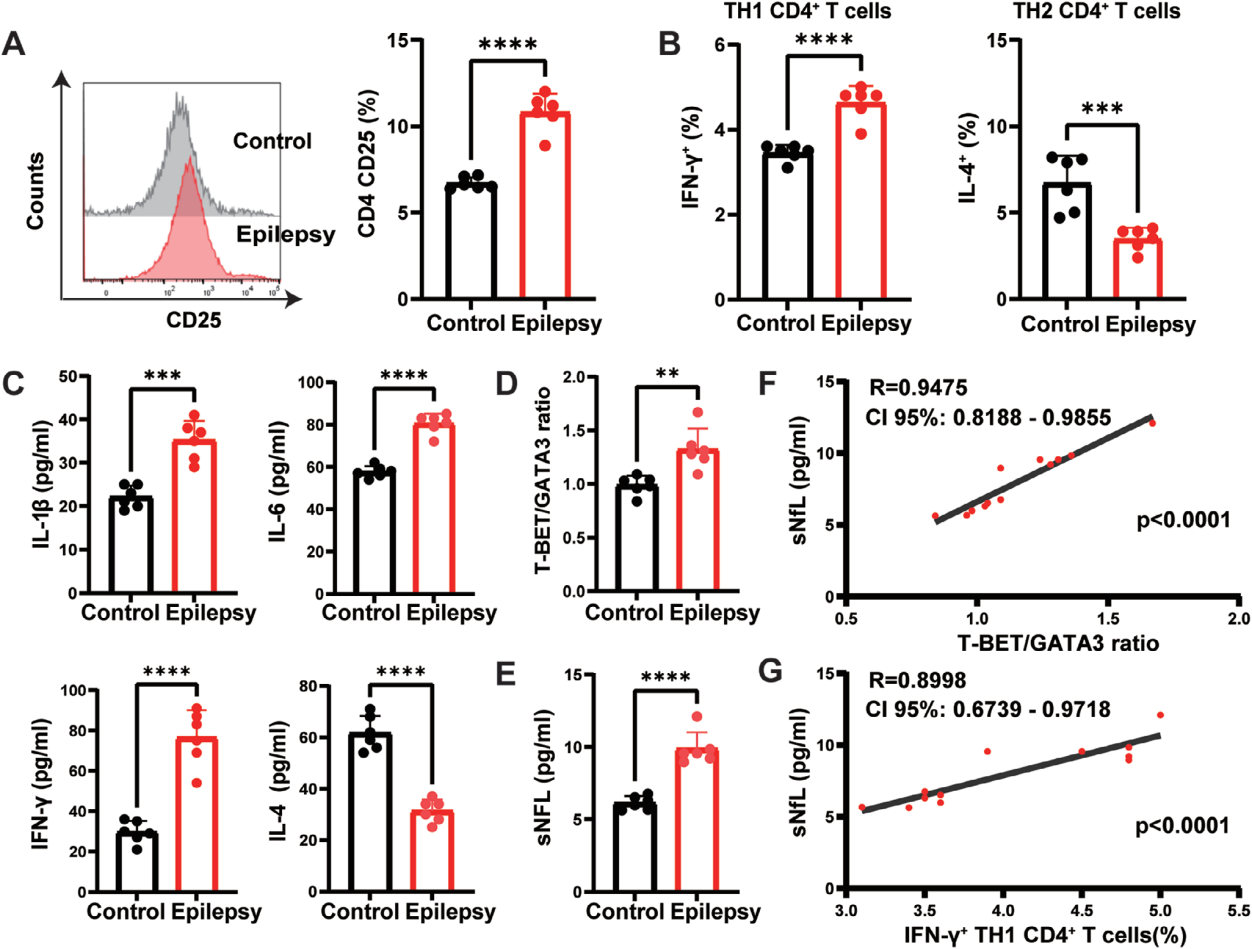
CD4^+^ T cells in the peripheral blood from lithium chloride‐pilocarpine temporal lobe epilepsy (TLE) rat models were activated and skewed toward a proinflammatory phenotype and associated with an increased concentration of sNfL. A) Representative MFI curves and levels of activation marker CD25 on the surface of CD4^+^ T cells from the TLE rats. B) Expression of Th1‐associated cytokines (IFN‐γ) and Th2‐related cytokine (IL‐4) in CD4^+^ T cells measured by flow cytometry. C) Levels of cytokines (IL‐6, IFN‐γ, IL‐1β, and IL‐4) in the serum from TLE rats determined by ELISA. D) The ratio of T‐bet and GATA3 determined by real‐time PCR. E) Concentration of sNfL in the serum from TLE rats detected by ELISA. Pearson correlation of sNfL concentration with T‐bet/GATA3 ratio F) and the proportion of IFN‐γ^+^ Th1 CD4^+^ T cells(G) in TLE rats. n = 6 per group. Data are presented as mean ± standard deviation. *P<0.05, **P<0.01, ***P<0.001, ****P<0.0001.

In the chronic stage of TLE rat models induced by lithium chloride‐pilocarpine, we also observed that CD4^+^ T cells were activated and tended to differentiate into the Th1 phenotype. Consistently, the levels of proinflammatory cytokines IL‐1β, IL‐6, and IFN‐γ were increased and that of anti‐inflammatory cytokine IL‐4 was decreased. In addition, the expression of sNfL was increased and associated with the Th1 CD4^+^ T cell and T‐bet/GATA3 ratio (**Figure** **2**).

### 2.3 CD4^+^ Th1 Polarization in the Epileptogenic Focus and Distant Brain Regions of TLE Rats

**Figure 3:**
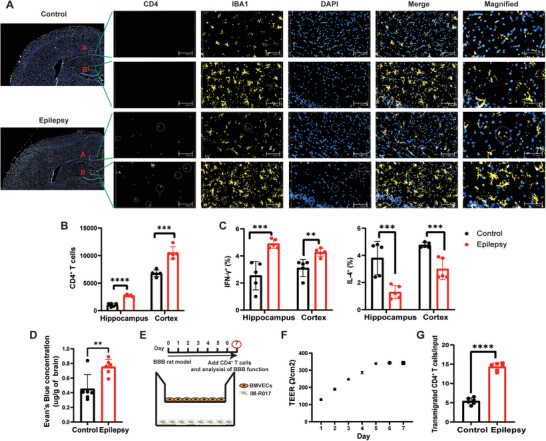
Peripheral CD4^+^ T cells infiltrate into the brain across the disrupted blood‐brain barrier (BBB). A) Representative immunofluorescence imaging in lithium chloride‐pilocarpine temporal lobe epilepsy rat models and normal control rats. The image shows CD4 (green), IBA1 (yellow), DAPI (blue) fluorescent signals, the merged signals, and the high‐resolution image of the circled areas in the hippocampus and cortex of brain. A represents cortex area and B represents hippocampus. The white rectangle indicates the magnified area. n = 3 per group. B) CD4^+^ T cell counts in the hippocampus and cortex of TLE rats determined by flow cytometry. C) Th1‐associated (IFN‐γ) and Th2‐related (IL‐4) cytokine levels in CD4^+^ T cells from brain tissue measured by flow cytometry. n = 5 per group. D) Evaluation of BBB permeability using Evans blue in the hippocampus and cortex of TLE rats and normal control rats. Evan's blue concentration was detected. n = 6 per group. E) Schematic diagram of in vitro BBB models. F) The time‐dependent progression of transendothelial electrical resistance (TEER) of the in vitro BBB model's cell monolayers was measured. G) Transmigration assay of the CD4^+^ T cells from the TLE rats and normal control rats across the BMVECs. The graph shows the percentage of transmigrated CD4^+^ T cells. n = 6 per group. Data are presented as mean ± standard deviation. *P<0.05, **P<0.01, ***P<0.001, ****P<0.0001.

To identify the interaction between the CD4^+^ T cells and central nervous system, we first determined whether circulating CD4^+^ T cells had infiltrated the brain parenchyma. Compared with normal control rats, we observed increased CD4^+^ T cell infiltration in rats with TLE, and the infiltrated CD4^+^ T cells were in close proximity to microglia (**Figure** **3**A,B). Furthermore, we performed flow cytometric characterization of resected brain tissue from the rat TLE model. Consistently, Th1 CD4^+^ T cells were present in a higher proportion in the hippocampus, which is considered the epileptogenic focus of this model. In contrast, the proportion of Th2 CD4^+^ T cells was decreased in the lithium chloride‐pilocarpine TLE rat model. Even in distant brain regions, we observed a shift toward Th1 polarization during the chronic phase of the model (Figure 3C).

### 2.4 Migration of Peripheral CD4^+^ T Cells Across the Disrupted Blood–Brain Barrier (BBB)

To understand how peripheral CD4^+^ T cells migrate into the brain parenchyma, Evans blue dye was injected intravenously into the tail vein of rats to quantitatively evaluate BBB permeability. Compared to normal control rats, the fluorescence intensity of Evans blue dye was increased in the brain tissue of rats with TLE (Figure 3D). Thus, we assumed that CD4^+^ T cells infiltrated the brain parenchyma across the disrupted BBB. We established an in vitro BBB model and reproduced BBB disruption (Figure 3E). During the development of this model, the transendothelial electrical resistance (TEER) was increased (Figure 3F), which may indicate an increased “looseness” of the monolayer. On the seventh day, freshly isolated CD4^+^ T cells from TLE and normal control rats were added to the upper chamber of the in vitro BBB model. The CD4^+^ T cell transmigration rate was measured to evaluate BBB disruption. Compared to the normal control group, the CD4^+^ T cell transmigration rate was significantly increased in the epilepsy group (Figure 3G).

### 2.5 CD4^+^ T Cells Regulate Microglia State Dynamics In Vitro and In Vivo

**Figure 4:**
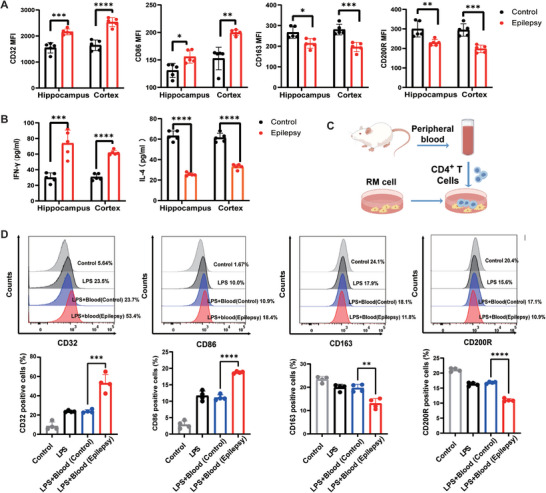
Microglia in the epileptogenic focus and distant cortex of TLE rats react to epilepsy, and CD4^+^ T cells affected microglial polarization in an in vitro LPS‐induced inflammatory model. A) Expression of CD32^+^/CD86^+^ and CD163^+^/CD200R^+^ microglia measured by flow cytometry in the hippocampus and cortex of TLE rats. B) Pro‐inflammatory cytokine (IFN‐γ) and anti‐inflammatory cytokine (IL‐4) levels determined by ELISA in the hippocampus and cortex of TLE rats. n = 5 per group. C) Flow diagram. D) The effect of CD4^+^ T cells on responding microglia in the lipopolysaccharide (LPS)‐induced microglial inflammatory model. The graph shows the proportions of CD32^+^/CD86^+^ and CD163^+^/CD200R^+^ microglia. Data are presented as mean ± standard deviation. *P<0.05, **P<0.01, ***P<0.001, ****P<0.0001.

Microglia are brain‐resident immune cells that are involved in the development of epilepsy. In the resected brain tissue of lithium chloride‐pilocarpine‐induced TLE rats in the chronic phase, we observed that the expression of CD32 and CD86 (markers of pro‐inflammatory microglia reactive to epilepsy) increased, and the expression of CD163 and CD200R (markers of anti‐inflammatory microglia reactive to epilepsy) decreased in the epileptogenic focus and distant brain regions (**Figure** **4**A). Consistently, the levels of IFN‐γ in the tissue homogenates of hippocampus and cortex were elevated, whereas the levels of IL‐4 were decreased (Figure 4B). Using immunofluorescence, CD4^+^ T cells were confirmed to be in close proximity to microglia (Figure 3A). Previous studies have demonstrated that CD4^+^ T cells influence microglial function. In vitro, we established Lipopolysaccharide (LPS)‐induced rat microglia (RM) cell inflammation models and added peripheral extracts of CD4+ T cells from TLE rats and controls (Figure 4C). Compared with the LPS^+^ blood (control) group, the CD32^+^/CD86^+^ microglial markers were upregulated in the LPS^+^ blood (seizure) group. In contrast, CD163^+^/CD200R^+^ microglial markers were downregulated (Figure 4D).

**Figure 5:**
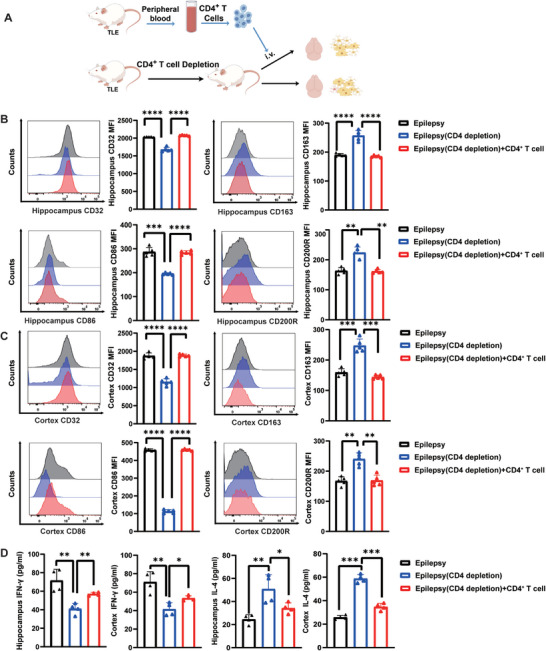
Intravenous depletion and reinfusion of CD4^+^ T cells regulate the microglial responses in the rat models with TLE. A) Flow diagram. Representative MFI curves and expression of markers associated with CD32^+^/CD86^+^ microglia and CD163^+^/CD200R^+^ microglia in the hippocampus B) and cortex C) of the epilepsy group, epilepsy with CD4^+^ T cells depletion group, and epilepsy with CD4^+^ T cells reinfusion group. n = 5 per group. D) The levels of pro‐inflammatory (IFN‐γ) and anti‐inflammatory (IL‐4) cytokines measured by ELISA in the hippocampus and cortex. n = 4 per group. Data are presented as mean ± standard deviation. *P<0.05, **P<0.01, ***P<0.001, ****P<0.0001.

We depleted CD4^+^ T cells in vivo by intravenously infusing anti‐CD4 antibody into the TLE rat model (**Figure** **5**A). After depletion of CD4^+^ T cells, the number of CD32^+^/CD86^+^ microglia in the hippocampus and cortex were decreased, whereas that of CD163^+^/CD200R^+^ microglia were increased. Upon venous reinfusion of CD4^+^ T cells extracted from TLE rats, the number of CD32^+^/CD86^+^ microglia in the hippocampus and cortex recovered, whereas that of CD163^+^/CD200R^+^ microglia decreased (Figure 5B,C). Furthermore, depletion of CD4^+^ T cells inhibited the secretion of pro‐inflammatory cytokine IFN‐γ and promoted the release of anti‐inflammatory cytokine IL‐4. Thus, the venous reinfusion of CD4^+^ T cells may accelerate the generation of IFN‐γ and reduce the production of IL‐4 (Figure 5D).

### 2.6 Blockage of Notch1 Signaling in the Periphery Regulates the Th1/Th2 Balance of CD4^+^ T Cells and the Microglia State Dynamics

**Figure 6:**
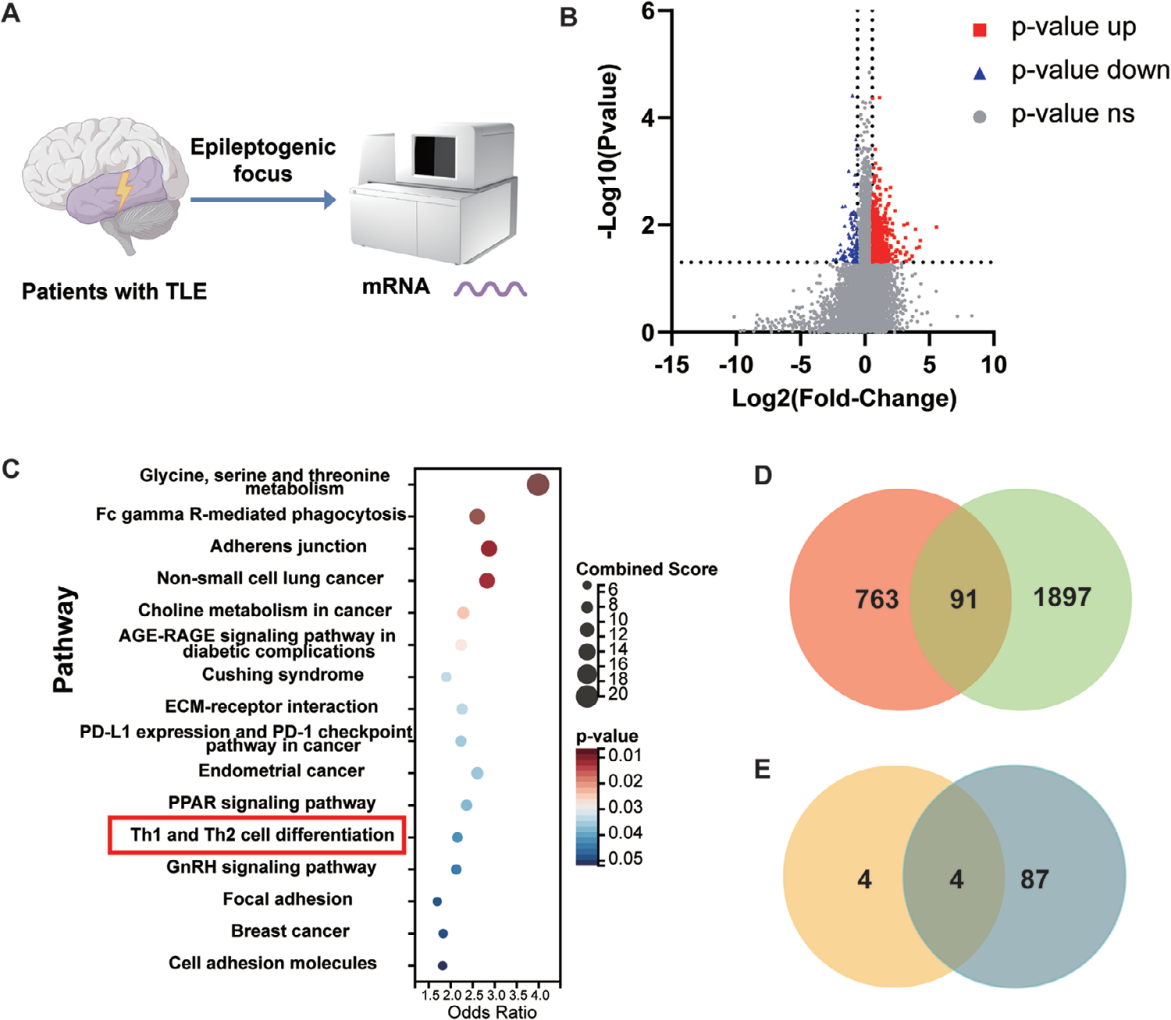
Differentially expressed genes in the hippocampus of patients with TLE were enriched in Th1 and Th2 cell differentiation genes by mRNA sequencing and bioinformatics analysis. A) Schematic diagram of mRNA sequencing of resected hippocampus from patients with TLE. B) Volcano plot of differentially expressed genes in hippocampal resections from patients with TLE. C) Bubble diagram of KEGG enrichment analysis of differentially expressed genes in hippocampal resections from patients with TLE. D) Venn diagram between the differentially expressed genes in patients with TLE and chemokines. E) Venn diagram between the differentially expressed chemokines in patients with TLE and hippocampal differentially expressed genes that are involved in Th1/Th2 differentiation.

**Figure 7:**
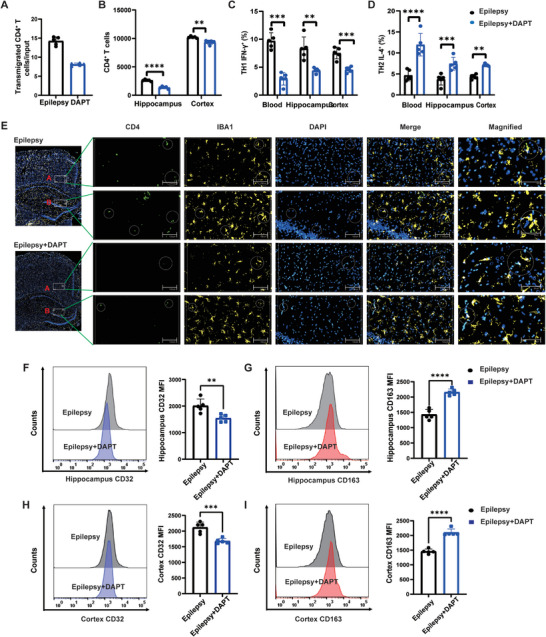
Notch 1 inhibitor DAPT reverses Th1/Th2 imbalance and modulates microglia states. A) Transmigration assay of the CD4^+^ T cells from the TLE rats and TLE rats treated with DAPT across the BMVECs in the in vitro BBB model. B) CD4^+^ T cell counts in the hippocampus and cortex of TLE rats treated with DAPT determined by flow cytometry. C) Th1‐associated (IFN‐γ) and D) Th2‐related (IL‐4) cytokine levels in CD4^+^ T cells extracted from the peripheral blood, hippocampus, and cortex of TLE rats treated with DAPT, measured by flow cytometry. n = 5 per group. E) Representative immunofluorescence imaging of brain of TLE rats and TLE rats treated with DAPT. n = 3 per group. Representative MFI curves and expression of M1 microglia marker CD32 F) and M2 microglia marker CD163 G) in the hippocampus measured by flow cytometry. Representative MFI curves and expression of M1 microglia markers CD32 H) and CD163 I) in the cortex measured by flow cytometry. n = 5 per group. Data are presented as mean ± standard deviation. *P<0.05, **P<0.01, ***P<0.001, ****P<0.0001.

To further search for crucial molecular targets to treat epilepsy, brain tissues were acquired from six patients with TLE who underwent surgical treatment, and mRNA sequencing of the resected epileptogenic foci was performed (**Figure** **6**A). In total, 854 differentially expressed genes (DEGs) were screened (Figure 6B), which were enriched in the Th1/Th2 cell differentiation pathway (Figure 6C). Ninety‐one DEGs might play a role in the chemotactic process (Figure 6D), among which four participate in Th1/Th2 cell differentiation (Figure 6E). As Notch1 primes CD4^+^ T cells for Th1 differentiation and may influence the entry of CD4^+^ T cells into the CNS, we further selectively inhibited Notch 1 using the gamma‐secretase inhibitor DAPT and found inhibition of Notch 1 lowered the number of migrated CD4^+^ T cells in the in vitro BBB model and reduced the capability of CD4^+^ T cells to cross the BBB (**Figure** **7**A). In the hippocampus and cortex, DAPT reduced CD4^+^ T cell infiltration (Figure 7B). Furthermore, DAPT reversed the Th1/Th2 imbalance of CD4^+^ T cells and promoted a shift to the Th2 subtype in the periphery and CNS (Figure 7C,D). In addition, the spatial distance between CD4^+^ T cells and microglia was increased (Figure 4E). In the hippocampus and cortex, DAPT decreased the number of CD32^+^/CD86^+^ microglia and increased that of CD163^+^/CD200R^+^ microglia (Figure 7F–I; Figure S3, Supporting Information).

**Figure 8:**
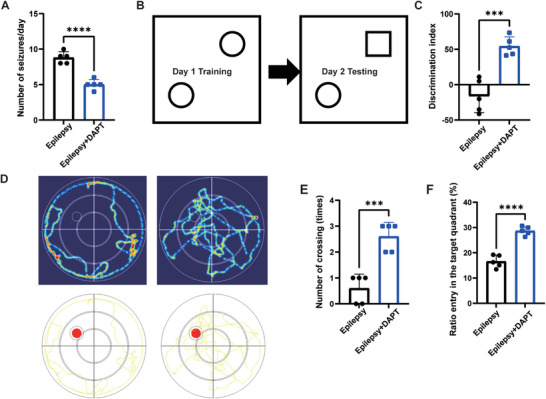
DAPT reduces seizure frequency and improves cognition in rats with TLE. A) The average number of seizures per day in rats. B) Novel object recognition (NOR) test. In the testing phase, the circle is a familiar object, whereas the square is a novel object. C) The discrimination indices for NOR tests of rats with TLE or those treated with DAPT. Number of times rats crossed the target location D) and the percentage of time spent in the target quadrant E) in the Morris water maze test. F) Swimming traces of rats in the probe trial. n = 5 per group. Data are presented as mean ± standard deviation. *P<0.05, **P<0.01, ***P<0.001, ****P<0.0001.

To confirm if DAPT could relieve seizures, the rats were monitored by video throughout the day for one week. We found that DAPT markedly reduced seizure frequency in rats with TLE (**Figure** **8**A). To determine whether DAPT can improve cognitive function in rats with TLE, we performed the novel object recognition (NOR) test to measure non‐spatial recognition memory (Figure 8B). As shown in Figure 8C, DAPT significantly improved the ability of the rats with TLE to recognize new and familiar objects compared with the untreated rats. To identify the effect of DAPT on spatial learning memory, the Morris water maze test was performed, and swimming traces of rats in the different groups are shown in Figure 8D. Compared to untreated rats with TLE, epileptic rats treated with DAPT showed more crossings through the target site and longer dwell time in the target quadrant (quadrant IV), suggesting the recovery of memory retention in rats with TLE treated with DAPT (Figure 8E,F).

## 3 Discussion

Th1 cells are known for their association with cell‐mediated immunity and pro‐inflammatory responses, whereas Th2 cells are associated with antibody‐mediated immunity and anti‐inflammatory effects. Numerous studies have underscored the involvement of both Th1 and Th2 responses in epilepsy. Importantly, the Th1/Th2 phenotype is indicative of T‐cell activation and the pro‐epileptogenic nature of the immune response, warranting attention. In our study involving patients with TLE, we observed activated CD4^+^ T cells in the periphery, with a noteworthy shift in the Th1/Th2 phenotype toward a proinflammatory Th1 predominance. This shift correlated with elevated levels of proinflammatory cytokines (IFN‐γ and TNF‐α), an increased T‐bet/GATA3 ratio, and heightened expression of the neuronal injury biomarker, sNfL. These findings suggest that peripheral Th1 cells may play a significant role in the peripheral immune response in patients with epilepsy. To investigate the underlying mechanisms, we used a classic preclinical animal model of acquired TLE. Alongside the consistent activation of peripheral CD4^+^ T cells and the Th1/Th2 phenotype observed in patients with TLE, we identified Th1 polarization in the epileptogenic focus, specifically in the hippocampus, which was later confirmed to infiltrate the highly permeable BBB. By employing the depletion and subsequent reinfusion of CD4^+^ T cells isolated from TLE rat plasma, we demonstrated that the Th1/Th2 phenotype mirrors the central microglia state dynamics. Proinflammatory Th1 cells notably influenced the responses of CD32^+^/CD86^+^ microglia in the hippocampus and cortex, thereby modulating central inflammation and contributing to the development of chronic recurrent seizures. Finally, our investigation revealed that blocking Notch1, identified through mRNA sequencing and specific to Th1 differentiation, could attenuate the pro‐inflammatory process and response during TLE. In summary, our research establishes a correlation between the imbalance of peripheral Th1/Th2 cells and central microglia state dynamics, shedding light on the coordinated response of the peripheral and central immune systems to epileptogenic or pro‐epileptogenic processes. Notably, we pinpoint Notch 1 as a promising therapeutic target by modifying the balance of the “systemic‐central” immune response and protecting against chronic neuronal injury during TLE.

### 3.1 Peripheral Th1/Th2 Imbalance Resonating with Central Microglia State Dynamics

Central and general inflammation are anatomically distinct phenomena; however, peripheral CD4^+^ T cells play a predominant role in epileptogenesis. Our study underscores the pivotal involvement of the Th1/Th2 imbalance in epilepsy pathogenesis, revealing a significant increase in the number of Th1 cells and a corresponding decrease in Th2 cells. While the current literature predominantly associates Th1 cells with inflammation in conditions such as TMEV‐induced seizures, West syndrome, and neurocysticercosis with seizures,^[^ ^23^ ^]^ limited research has explored alterations in the Th1/Th2 paradigm specific to epilepsy. To date, only one study has proposed a significant Th1/Th2 signature pattern in epilepsy, which conflicts with our observations. Hou et al. conducted a single‐sample gene set enrichment analysis and gene set variation analysis on normal and epilepsy samples, suggesting a shift from Th1/Th2 balance.^[^ ^24^ ^]^ Notably, this discrepancy may stem from differences in research subjects. Our study maintained higher homogeneity, focusing exclusively on TLE in both human tissues and preclinical models, whereas Hou et al. incorporated highly heterogeneous epilepsy samples, including patients with tuberous sclerosis complex, primary brain tumors, and refractory epilepsy. Despite this variation, both studies emphasized the pivotal role of the Th1/Th2 paradigm in the inflammatory response during epilepsy. This controversy underscores the involvement of multiple complex regulators of the Th1/Th2 imbalance in epilepsy and suggests that the Th1/Th2 signature may respond differently in diverse types of epilepsy. Additionally, our study revealed an intriguing parallel between the Th1/Th2 and microglia state dynamics in the epileptic brain. In the preclinical model, both the Th1/Th2 paradigm and microglia state dynamics, which serve as proinflammatory and anti‐inflammatory representatives in the peripheral and central compartments, respectively, exhibit similar response patterns during epilepsy. Similar crosstalk between T cells and microglia has been observed in studies on cerebral ischemia, where Th1/CD86^+^microglia crosstalk exacerbates immune responses after stroke, contributing to brain damage, whereas crosstalk between Th2 and CD163^+^microglia plays an anti‐inflammatory role, contributing to brain recovery.^[^ ^25^, ^26^ ^]^ Furthermore, our findings indicated that the intervention of peripheral CD4^+^ T cells can affect the reaction of central microglia. This suggests that the Th1/Th2 paradigm not only serves as a potential biomarker reflecting the extent and direction of central inflammation but also represents an intervention target for inflammation in TLE.

### 3.2 Deciphering the Intricacies of the General‐Central Inflammation Network

The echo phenomenon between Th1/Th2 phenotypes and microglia state dynamics represents only one facet of the intricate interplay between general and central inflammation in response to TLE. Our study navigated the complexities of the “general‐central inflammation” network, uncovering additional dimensions that contribute to a more comprehensive understanding of this intricate interaction.

First, focusing on general inflammation, our investigation revealed a notable increase in peripheral CD4^+^ T cells in both rat and human plasma samples. Within this cell population, the Th1 subset demonstrated prominence, accompanied by an elevation in pro‐inflammatory cytokines. This finding aligns with the research of Ouédraogo et al. research, indicating proclivity for a pro‐inflammatory phenotype in circulating CD4^+^ T cells, along with increased levels of pro‐inflammatory cytokines.^[^ ^14^ ^]^ Furthermore, the ratio of T‐bet (a transcription factor associated with Th1 cells) to GATA3 (a transcription factor linked to Th2 cells), an indicator of the potential transformation of CD4^+^ T cells,^[^ ^27^ ^]^ was remarkably increased in individuals with TLE. This observation signified a heightened propensity of these cells to adopt the Th1 subtype following an epileptic insult. Notably, the proportion of CD4^+^ T cells and elevated T‐bet/GATA3 ratio showed a positive correlation with soluble neurofilament light chain (sNfL) levels, implying a potential link between the response of circulating CD4^+^ T cells and neuronal injury during TLE development and implicating these cells in the pathophysiology of epilepsy‐related neuronal damage.

Second, beyond the observed increase in CD4^+^ T cells in the periphery post‐TLE, our study revealed a heightened presence of pro‐inflammatory CD4^+^ T cells within the brain tissues of rats experiencing TLE, which is consistent with previous research. Under normal conditions, a certain proportion of CD4^+^ T‐cells are present in the CNS. However, under pathological conditions, peripheral T‐cells traverse the BBB and infiltrate the CNS, contributing to disease progression.^[^ ^28^ ^]^ Our preclinical model revealed that pro‐inflammatory CD4^+^ T cells preferentially penetrated the BBB in response to the onset of epilepsy, even in the absence of peripheral inflammation. This observation aligns with existing research, and we propose that a substantial influx of CD4^+^ T cells may migrate into the CNS to counteract the inflammation triggered by seizures.

Lastly, our investigation revealed the close proximity of CD4^+^ T cells to microglia in both the hippocampus and cortex following TLE, suggesting an interactive relationship between infiltrating CD4^+^ T cells and microglia, with implications for pro‐epileptogenesis. Previous research has demonstrated that the depletion of CD4^+^ T cells in healthy rodents or humans can lead to an abnormal microglial phenotype and synapse overactivity.^[^ ^29^ ^]^ In our study, we confirmed that depleting and subsequently reinfusing CD4+ T cells in the plasma of TLE rats regulates microglial state dynamics, impacting the secretion of IFN‐γ and IL‐4, influencing the inflammatory response in both epileptic loci and the cortex. Consequently, the infiltration of CD4^+^ T cells into the CNS influences pro‐epileptic central inflammation by microglia in response to epilepsy. In summary, our thorough investigation elucidated the intricate dynamics of the “general‐central inflammation” network, highlighting the pivotal role played by peripheral CD4^+^ T cells and their subtype phenotypes in echoing microglia‐related inflammation in the CNS, transversing the BBB and engaging in physical interaction with microglia. Further exploration is imperative to elucidate the detailed molecular mechanisms underlying these complex interactions.

### 3.3 A Potential Specific Target at the Periphery for TLE Inflammatory Management

Notch1, a transmembrane receptor, plays a pivotal role in immune response. Previous studies have proposed that Notch signaling promotes Th1 cell differentiation while concurrently inhibiting Th2 cell differentiation.^[^ ^30^ ^]^ Notably, the activation of Notch 1 has been linked to the upregulation of T‐bet and Th1 polarization thorough Akt/ERK‐GSK3β signaling axis.^[^ ^31^ ^]^ GATA3 also regulates human T‐cell lineage commitment by restraining Notch activity.^[^ ^32^ ^]^ These findings underscore the significance of Notch 1 signaling in T‐cell differentiation, tilting the balance toward a Th1‐type immune response. Beyond its role in the periphery, Notch signaling has been implicated in microglia‐mediated central inflammation in ischemic stroke and multiple sclerosis,^[^ ^33^, ^34^ ^]^ in either human or experimental models. Using sequencing and bioinformatics to examine the resected hippocampal tissue of individuals with TLE, we identified significant changes in gene expression in the Th1/Th2 differentiation pathway. Notably, Notch 1 is a key regulator of the transition of CD4^+^ T cells into the Th1 subtype and plays a role in recruiting CD4^+^ T cells within the CNS. Interestingly, experimental inhibition of Notch signaling through intravenous administration of DAPT in the TLE rat model yielded promising results. Specifically, we observed a substantial reduction in the infiltration of CD4^+^ T cells into the CNS, along with the suppression of microglial polarization toward the anti‐inflammatory phenotype. These findings provide compelling evidence of the critical role of Notch in the intricate interplay between CD4^+^ T cells and microglia in TLE. Therefore, we believe that DAPT has broad prospect and Notch 1 inhibitor may become a novel prospective therapeutic agent for epilepsy or other central autoimmune diseases also with systemic‐central inflammation crosstalk. But more details including intervention point and optimal dosages should be further addressed. Meanwhile, we had to admit that Notch1 also has been identified participating in the regulation of central neurogenesis or neuroplasticity, thus we cannot rule out their possible influences on epilepsy via other signaling pathways and mechanisms. In our study, we did not perform EEG recordings because the procedure significantly damages the BBB and leads to inflammatory responses, and thus, bias when examining the immune responses in the brain.

Furthermore, in our current study, we conducted preliminary assessments using Morris water maze (MWM) and novel object recognition (NOR) tests to evaluate spatial and object recognition memory. Our findings suggested that DAPT administration improved cognition in rats with epilepsy. Additionally, we assessed the frequency of convulsive seizures using the Racine scale and observed a preliminary decrease in seizure frequency following DAPT administration. However, to validate this trend more accurately, further investigation utilizing more specific and objective methods such as 24‐hour video‐electroencephalography (EEG) is warranted.

## 4 Conclusion

In summary, our investigation highlights the intricate association between TLE and the related networks of general and central inflammation. This inflammatory response encompasses the migration of peripheral pro‐inflammatory CD4^+^ T cells across a compromised BBB and their subsequent infiltration into the CNS, with specific targeting of the epileptic loci and cortex. The crucial roles played by Th1/Th2 cells in the periphery reflect the microglia state dynamic in the CNS, and the ensuing interaction between these CD4^+^ T cells and microglia significantly contributes to the inflammatory response driving the progression of epilepsy. Importantly, our findings underscore the pivotal role of Notch1 in orchestrating microglia state dynamics and influencing the Th1/Th2 differentiation of CD4^+^ T cells. Given its central involvement in these processes, Notch1 has emerged as a potential therapeutic target for alleviating the effects of central inflammation in TLE. This study has some limitations. First, our results were specific to temporal lobe epilepsy; other types of epilepsy may elicit different inflammatory responses, necessitating further investigation. In addition, employing different TLE‐related preclinical animal models could help replicate and validate our observations. Second, our focus on the predominant subtypes of CD4^+^ T cells, namely Th1 and Th2, should be expanded in future studies to encompass a broader array of subtypes. Lastly, our selection of 2–3 cytokines related to Th1 or Th2 is a partial representation, and future studies could incorporate a more extensive panel of cytokines and chemokines to establish a more intricate regulatory network between the peripheral and central inflammatory systems.

## 5 Experimental Section

### Subjects

This study enrolled 27 patients with temporal lobe epilepsy who were undergoing treatment at Xiangya Hospital, Central South University, from December 01, 2022, to January 31, 2023. By assessing their medical history, EEG, and imaging data, two trained epileptologists diagnosed these patients with temporal lobe epilepsy according to the diagnostic manual of the International League Against Epilepsy. These patients were 18–70 years old and seizure‐free for at least 72 h. The exclusion criteria were as follows: 1) active neoplasm, 2) autoimmune diseases, 3) infectious disorders, 4) cachexia, and 5) pregnancy. Twenty‐seven age‐ and sex‐matched healthy controls were recruited for this study. Informed consent was obtained from all participants and all procedures were approved by the Ethics Committee of Xiangya Hospital, Central South University (2 022 020 273).

### Lithium Chloride‐Pilocarpine Temporal Lobe Epilepsy Rat Model

Adult male Sprague–‒Dawley rats (weight: 180–220 g) were acquired and raised at the Department of Laboratory Animals, Central South University. All animal experimental protocols were approved by the Experimental Animal Ethics Committee of Xiangya Hospital, Central South University. After a week of adaptive feeding, the rats were injected with lithium chloride (125 mg kg^−1^, Dingguo, China). After 18–24 h, racemic hyoscyamine hydrochloride (1 mg kg^−1^, Department of Pharmacy, Xiangya Hospital, Central South University) was administered. Thirty minutes later, the rats in the experimental group were intraperitoneally injected with pilocarpine (25 mg kg^−1^, Sigma, USA) to induce status epilepticus (SE), and rats injected with saline were considered normal controls. The behaviors of the rats were observed and scored using a modified Racine scale. Thereafter, an additional 12.5 mg kg^−1^ dose of pilocarpine was administered every 30 min until the rats developed status epilepticus (SE) with no intermittent episodes above the Racine score of IV‐V. Sixty minutes after SE, all rats were intraperitoneally administered diazepam (10 mg kg^−1^; Xiangya Hospital, Central South University, China) to stop SE.

### Animal Model Preparation

The sample size calculation was performed in advance for the animal studies using Resource Equation Approach. For an independent t‐test, the between‐subject error DF (that is, the within‐subject DF) is calculated as: DF = N – k = kn – k = k (n – 1). Based on the acceptable range of the DF, we obtained the minimum numbers of each group of TLE rats is ≈6. In our experiments, total of 110 male Sprague‐Dawley rats (weight: 180–220 g) was used for pilocarpine‐induced SE, and 83 rats were survived with the mortality of 25%. Only animals during the chronic phase (60 d after SE) that presented recurrent seizures were used for further experiments. Rat models were randomly divided into 7 groups: control (n = 19), epilepsy (n = 19), epilepsy (depletion control) (n = 6), epilepsy (CD4+T depletion) (n = 6), epilepsy (CD4+T depletion +CD4+T cell) (n = 6), epilepsy+sham (n = 13) and epilepsy+DAPT (n = 13). Among them, 24 model rats in total were observed for Immunofluorescence and Flow cytometry separately, and another 55 rats were used for Flow cytometry. The TLE rats were euthanized for tissue collection and other evaluations in chronic period which defined as the stage that 60 days after status epilepticus induced by lithium chloride‐pilocarpine accompanied with recurrent seizures in animals.

### Evans Blue Dye

During the chronic stage, lithium chloride‐pilocarpine temporal lobe epilepsy rats were anesthetized with sodium pentobarbital. 2% (w/v) Evans blue dye solution (3 ml kg^−1^) was injected intravenously into the tail vein of the rats and allowed to circulate for one hour. During this period, the animals were allowed to wake up. One hour later, rats were anesthetized again and transcardially perfused with 0.9% normal saline. Brain tissue was then dissected and stored at −80 °C until analysis. Brain tissue was homogenized with 0.01 M PBS. The tissue homogenates were then centrifuged for 20 min at 1000× g. The preserved supernatants were mixed with dimethylacetamide (Solarbio, D6060 1 mL/100 mg), and the fluorescence intensity was measured at 620 nm. An Evans blue standard curve was used to convert the absorbance values to ng dye.

### In Vitro BBB Model and Migration Assay

An in vitro BBB model was established as previously described. Rat brain microvascular endothelial cells (BMVECs, CP‐R108) were cultured on a 0.4‐µm pore filter (polycarbonate membrane of Transwell 24‐well cell culture chamber, N725101). On the same day, CTX‐TNA2 Rat Brain Type I Astrocytes (IM‐R017) were cocultured in the lower chamber. The transendothelial electrical resistance (TEER) of the cell monolayer was measured with an Endohm resistance meter (World Precision Instruments, FL, United States) to determine the resistance of tight junctions in in vitro rat BBB model. The TEER values of an empty filter were measured to calculate the net resistance of the cell monolayers, TEER (Ω*cm2) = (cell monolayer resistance− empty Transwell filter resistance) ×surface area (cm^2^). On the seventh day, CD4^+^ T cells (1.5×10*5 cells per assay) extracted from the normal and epileptic rats were added to the upper chamber and co‐cultured for 2 hours. The migrated CD4^+^ T cells in the lower chamber were counted using a cytometer.

### Cell Isolation of CD4^+^ T Cells

The TLE rats in the chronic phase were anesthetized, and peripheral blood was acquired by venipuncture from the retro‐orbital plexus. Peripheral blood mononuclear cells (PBMCs) were then isolated as previously described. PBMCs were incubated with PE anti‐rat CD4^+^ (domain2) (BioLegend, 203 307) for 20 min. CD4^+^ T cells were then magnetically isolated from the PBMCs using anti‐PE microbeads (Miltenyi Biotec, 130‐048‐801), according to the manufacturer's protocol.

### In VIVO depletion and Reinfusion of CD4^+^ T Cells in TLE Rats

TLE rats (60 d after SE) were intravenously injected with anti‐CD4 mAb (BioXCell, BE0308) to deplete CD4+ T cells, with a dose of 50 µg in the chronic phase once every other day for twice in total. IgG antibody (Rat IgG2b, BioXCell, BE0252) was used as control for anti‐CD4 mAb. In group of epilepsy (CD4 deletion+CD4+T cell) rats, the pre‐extracted CD4+ T cells would be injected back into tail vein of model rats after CD4+T deletion

### In Vitro Verification of the Effect of CD4^+^ T Cells on Microglia

Rat microglia (RM; BNCC360237) were cultured as described previously. Lipopolysaccharides (LPS) (10 µg ml^−1^) was used for 24 h to induce an inflammatory reaction. Then, microglia stimulated with LPS were added to CD4^+^ T cells (1.5×10*5 cells) isolated from normal rats and TLE rats. The microglial state was analyzed using flow cytometry.

### Flow Cytometry

For the analysis of CD4^+^ T cell activation by flow cytometry, single‐cell suspensions from the brain tissue and blood were prepared as follows: Briefly, rats were anesthetized and perfused through the left ventricle with ice‐cold 1X HBSS, and the hippocampus and cortex were removed. The brain tissues then were homogenized in PBS and strained through a 70‐µm cell strainer. The homogenates were centrifuged at 1800 rpm for 5 min. Peripheral blood of the rats was acquired by venipuncture from the retro‐orbital plexus, and the peripheral blood of patients with epilepsy was obtained from the ulnar vein. The PBMCs were extracted as previously described. The monocytes from brain tissue and PBMCs from blood of rats were incubated with the following antibody mixtures for 20 min in the dark at room temperature: bv421 anti‐rat CD3 (BD Biosciences, 563 948), FITC anti‐rat CD4 (BD Biosciences, 561 833), PE anti‐rat CD8 (biolegend, 201 705), APC anti‐rat CD25 (Thermo Fisher, 17‐0390‐82). PBMCs were incubated with the following antibodies: bv510 anti‐human CD3 (BD Biosciences, 563 109), bb515 anti‐human CD4 (BD Biosciences, 564 500), alexa fluor700 anti‐human CD8a (BD Biosciences, 561 026), bv785 anti‐human CD25 (BD Biosciences, 563 700), bv605 anti‐human HLA‐DR (BD Biosciences, 562 844), pe‐cy5 anti‐human CD69 (BD Biosciences, 310 907). To further analyze the intracellular cytokine expression levels, cell stimulation mixtures (with protein transport inhibitors) were added to the PBMCs for 4–6 h. Then, the cells from rats were permeabilized and stained using monoclonal antibodies for pe anti‐rat IL‐4 (biolegend, 511 906) and alexa fluor647 anti‐rat IFN‐γ (BD Biosciences, 562 213). The cells from patients were incubated with pe‐cy7 anti‐human IL‐4 (BD Biosciences, 560 672), bv421 anti‐human IL‐10 (BD Biosciences, 566 276), percp‐cy5.5 anti‐human IFN‐γ (biolegend, 502 525), PE‐dazzle594 anti‐human TNF‐α (biolegend, 502 945). The phenotype of the CD4^+^ T cells was analyzed using flow cytometry.

For analysis of the microglial state by flow cytometry, a single‐cell suspension was prepared from the hippocampus and cortex of rats as well as RM cells. The hippocampus and cortex were both minced and passed through a 70‐µm cell strainer. The homogenates were centrifuged at 1800 rpm for 5 min. Brain tissue monocytes were extracted as described previously. The extracted monocytes and RM cells were resuspended in 100 µl PBS with 0.1% bovine serum albumin (BSA) and incubated with the following antibody mixtures for 20 minutes in the dark at room temperature: APC/FireTM750 anti‐rat CD45 (BioLegend, 202 222), PE/Cy7 anti‐rat CD11b/c (BioLegend, 201 818), FITC anti‐rat CD86 (BioLegend, 200 305), mouse anti‐rat CD32 BV421 (BD Biosciences, 740 047), and mouse anti‐rat CD163 RPE (Bio‐Rad, MCA342PE), APC anti‐rat CD200 (eBioscience, 17 920 042). Finally, all the samples were resuspended in 200 µl PBS/BSA and immediately read using a Dxp Athena flow cytometer (Cytek, USA). The mean fluorescence intensity (MFI) of CD25, CD69 and HLR‐DR, CD32, CD86, CD163, CD200R, IL‐4, IL‐10, TNF‐α, and IFN‐γ were calculated using FlowJo V10.0 software (Tree Star, USA).

### Quantitative Real‐Time Polymerase Chain Reaction

mRNA was extracted from the peripheral blood of patients with epilepsy and rats with TLE. Total RNA (1 µg) was reverse transcribed using HiScript II Q RT SuperMix for qPCR (+gDNA wiper) kit (R223‐01, Vazyme Biotech Co., Ltd, Nanjing, China) according to the manufacturer's instructions. RT‐qPCR was performed using the ChamQ Universal SYBR qPCR Master Mix (Q711‐01, Vazyme Biotech Co., Ltd., Nanjing, China) on a StepOne Plus Real‐Time PCR System (Applied Biosystems, USA). The 2−ΔΔCT method was used, and glyceraldehyde‐3‐phosphate dehydrogenase (GAPDH) was selected as a reference gene to calculate the relative fold changes in mRNA expression. The following primer pairs were used: rat: T‐bet forward: CACTGGATGCGACAGGAAGT, and reverse: AGAGGGTAGGAATGTGGGCT; GATA3 forward: ACCACCTATCCGCCCTATGT and reverse: TTGAAGGAGCTGCTCTTGGG; GAPDH forward: GACATGCCGCCTGGAGAAAC and reverse: AGCCCAGGATGCCCTTTAGT; human: T‐bet forward: ATGATTGTGCTCCAGTCCC, and reverse: CCTCTGGCTCTCCGTCGTTC; GATA3 forward: GGCGCCGTCTTGATACTT and reverse: CTGGGTAGCGAAGAGCAGAG; GAPDH forward: CAGGAGGCATTGCTGATGAT and reverse: GAAGGCTGGGGCTCATTT.

### Immunofluorescence

In the chronic phase of the lithium chloride‐pilocarpine temporal lobe epilepsy rat model, rats were anesthetized and perfused through the heart. Phosphate‐buffered saline (0.1%) and 4% paraformaldehyde were added successively. After post‐fixation, dehydration, and embedding, the brain tissue was continuously sectioned into 15 µm slices on a cryostat. Three slices were randomly selected and incubated in citrate buffer (pH6.0) for 5 min. Then, the slices were blocked in 10% goat serum for 30 min, following incubation with rabbit anti‐Iba1 (Ab283319, abcam) and CD4^+^ antibody (Ab133616, abcam) overnight at 4 °C. The secondary antibodies were donkey anti‐mouse IgG H&L (FITC) (ab97029, abcam) and donkey anti‐rabbit IgG H&L (Alexa Fluor 555) (ab150075, abcam). After washing in PBS, the slices were sealed with mounting medium containing DAPI‐aqueous Fluoroshield (ab10413, abcam). The slices were imaged with a Nikon eclipse 80i microscope. Three to five random non‐overlapping views of each slice were selected, and the numbers of CD4^+^ T cells and Iba1^+^ cells were counted, and the distance between CD4^+^ T cells and Iba1^+^ cells was measured using ImageJ.

### Determination of Cytokine Production in the Peripheral Blood and Brain

The level of interleukins‐4 (IL‐4), IL‐6, and IL‐1β in serum from the peripheral blood of the patients with temporal lobe epilepsy and TLE rats, and the level of IFN‐γ and IL‐4 in homogenates from the cortex and hippocampus of the rats, were measured according to the manufacturer's protocol of commercially available ELISA kits (Abcam). Absorbance was measured at 450 nm on a Titertek ELISA reader, and the concentration of the cytokines was calculated using a standard curve constructed from continuous dilutions of standards.

### Serum Neurofilament Assay

The concentrations of sNfL in the sera of patients with epilepsy and TLE rats were measured using a Human Neurofilament protein L (NF‐L) ELISA kit (Cusabio, CSB‐E16094h) and a Rat Neurofilament light polypeptide (NEFL) ELISA kit (Cusabio, CSB‐EL015688RA), respectively, according to the manufacturers’ protocols.

### mRNA Sequence and Bioinformatics Analysis of Hippocampal Tissue from Patients with TLE and Autopsied Patients

Patients and Tissue Preparation: We enrolled six patients with TLE who underwent standard hippocampectomy at the Department of Neurosurgery of Xiangya Hospital. All patients were confirmed the diagnosis of temporal lobe epilepsy by their clinical manifestations, electrophysiology and MRI findings as well as postoperative pathological outcome. Informed consents were obtained from all patients. Samples of the control group were obtained from autopsied patients who joined in the cadaver donation program of Xiangya School of Medicine, without neuropsychiatric disorders and abnormalities in the central nervous system based on clinical history or histological examination. The samples obtained were snap‐frozen in liquid nitrogen and stored at −80 °C, then used for mRNA sequencing.

*Differentially expressed genes (DEGs)*: RNA‐seq data were first performed quality control by Trim Galore and mapped to hg19 by Hisat2 with its default parameters. The read counts for each gene were calculated by feature Counts with parameters ‐s 2 and ‐M. Differentially expressed genes (DEGs) were identified by DESeq2 with the cutoffs as abs(log2FoldChange) ≥0.58 and p‐value≤0.05.

*GO and KEGG Pathway Enrichment Analysis*: GO enrichment and KEGG pathway analysis were applied to systematically investigate the biological functions of selected DEGs using the Database for Annotation, Visualization, and Integrated Discovery (DAVID) version 6.829. P and FDR values < 0.05 were considered significantly different.

*Data Acquisition and Differentially Expressed Chemokine Related Genes*: To identify differentially expressed genes in chemotactic process, chemokine related genes were extracted from GeneCards (https://www.genecards.org) with a relevance score≥7, and further preprocessed with the limma package in view of a false discovery rate (FDR) < 0.05 and |log2 fold change (FC)| ≥ 1, in accordance with previously reported methods. Meanwhile, genes with an average count value of < 1 were eliminated. The overlapping genes between the above chemokine related genes and DEGs through mRNA sequencing were thus considered as key genes for chemotactic recruitment of peripheral immune cells into the brain.

### DAPT Administration

In the chronic stage of the model (60 d after SE), the TLE rats received an injection with a γ‐secretase inhibitor of Notch signaling, N‐[N‐(3,5‐difluorophenacetyl)‐L‐alanyl]‐S‐phenylglycine t‐butyl ester (DAPT, purchased from Selleck Chemicals, s2215, 10 mg kg^−1^) in the tail vein once daily for 1 week. The epilepsy+sham group was injected with an equal volume of saline.

### Behavioral Observations

All experimental animals were first numbered. After epilepsy model was successful established through kindling by lithium chloride‐pilocarpine, epilepsy+DAPT group and epilepsy+sham group were observed during the chronic phase of the model (60 d after SE). Behavioral testing and the occurrence of spontaneous recurrent seizures were detected and analyzed. Technicians performing behavioral assessment were blind to the treatment groups.

*Video Monitoring*: To identify the effect of DAPT on seizure frequency, TLE rats and models treated with DAPT were monitored by video for one week during the chronic stage. The number of seizures per day was calculated.

To assess the effect of DAPT on cognitive function in rats with TLE, the Morris water maze (MWM) and novel object recognition (NOR) tasks were performed.

*Morris water maze (MWM) test*: The maze consisted of a cylindrical pool and an image acquisition and analysis system. The pool was divided into four equal quadrants (I‐IV). Before training, the rats were allowed to swim freely in the pool (without a platform) for 2 min to familiarize themselves with the maze environment. Training was conducted four times per day for four days. The platform was placed in quadrant II. The rats were placed in the pool from four different quadrants in four training sessions and the time taken to find the platform was recorded. If the rats could not find the platform within 60 s, they were helped to find the platform. After resting on the platform for 10 s, the rats were trained again. The platform was then removed from the spatial probe trial. The rats were placed in the pool at the same points, and their swimming trajectories were recorded for 60 s. The number of times the rats crossed the platform, and the percentage of time spent in the target quadrant II were analyzed.

*Novel Object Recognition (NOR) Task*: The task was divided into three stages: adaptation, familiarity, and testing. During the adaptation period, the rats were placed in the experimental device and allowed to move freely for 10 min. Two identical objects were placed in the experimental device. The rats were placed in the device at an equal distance away from the object, with their backs facing the object, and the time spent exploring each object was recorded within 5 min. After one hour, one of the two identical objects was replaced with a different object, the rat was again placed with its back to the object at an equal distance from the object, and the time spent exploring each object was recorded within 5 min. The discrimination index (DI) was calculated. DI = (B − A)/(B + A). A represents the time spent with the familiar object and B represents the time spent with the novel object.

### Statistical Analysis

Continuous data were described using mean ± S.D. Data that passed normality test (Shapiro Wilks) were analyzed by independent samples t test or one way ANOVA with Tukey's multiple comparisons test. Correlation analysis was performed by Pearson's rank with GraphPad Prism v.9 or later, p < 0.05 was considered significant.

## Conflict of Interest

The authors declare no conflict of interest.

## Supporting information

Supporting Information

## Data Availability

The data that support the findings of this study are available from the corresponding author upon reasonable request.
