# Supplementary material for: Th1/Th2 Imbalance in Peripheral Blood Echoes Microglia State Dynamics in CNS During TLE Progression
Source: Adv Sci (Weinh). 2024 Aug 13;11(39):2405346. doi: 10.1002/advs.202405346 (PMC11496985; doi:10.1002/advs.202405346)
Supplement: Supplementary file 1 — Supporting Information [file ADVS-11-2405346-s001.docx]

**Supplementary Materials for**

**Th1/Th2 Imbalance in Peripheral Blood Echoes Microglia State Dynamics in CNS during TLE Progression**

**Authors:** Jing Wang^1,2†^, Yuanxia Wu^1,3†^, Jing Chen^4,^ Qiong Zhang^1,2^, Yunyi Liu^1,2^, Hongyu Long^1,2^, Jianhua Yu^6^，Qian Wu^5^*, Li Feng^1,2^*

**Affiliations:**

^1^Department of Neurology, Xiangya Hospital, Central South University; Changsha, Hunan, 410008, China.

^2^National Clinical Research Center for Geriatric Disorders, Xiangya Hospital, Central South University; Changsha, Hunan, 410008, China.

^3^Department of Neurology, Guizhou Provincial People's Hospital; Guiyang, Guizhou 550002, China.

^4^State Key Laboratory of Oral Diseases & National Center for Stomatology & National Clinical Research Center for Oral Diseases & Department of Operative Dentistry and Endodontics， West China Hospital of Stomatology, Sichuan University; Chengdu 610041, Sichuan, China

^5^Department of Neurology, First Affiliated Hospital, Kunming Medical University; Kunming 650032, Yunnan, China.

^6^Department of Immuno-Oncology, City of Hope; Los Angeles, CA 91010, USA.

*Corresponding author. Email: [fenglihx@163.com](mailto:fenglihx@163.com), [qianwu@ydyy.cn](mailto:qianwu@ydyy.cn) .

† These authors contributed equally to this work.

**This PDF file includes:**

Fig S1 to S3 for multiple supplementary figures

**Fig. S1.**

**
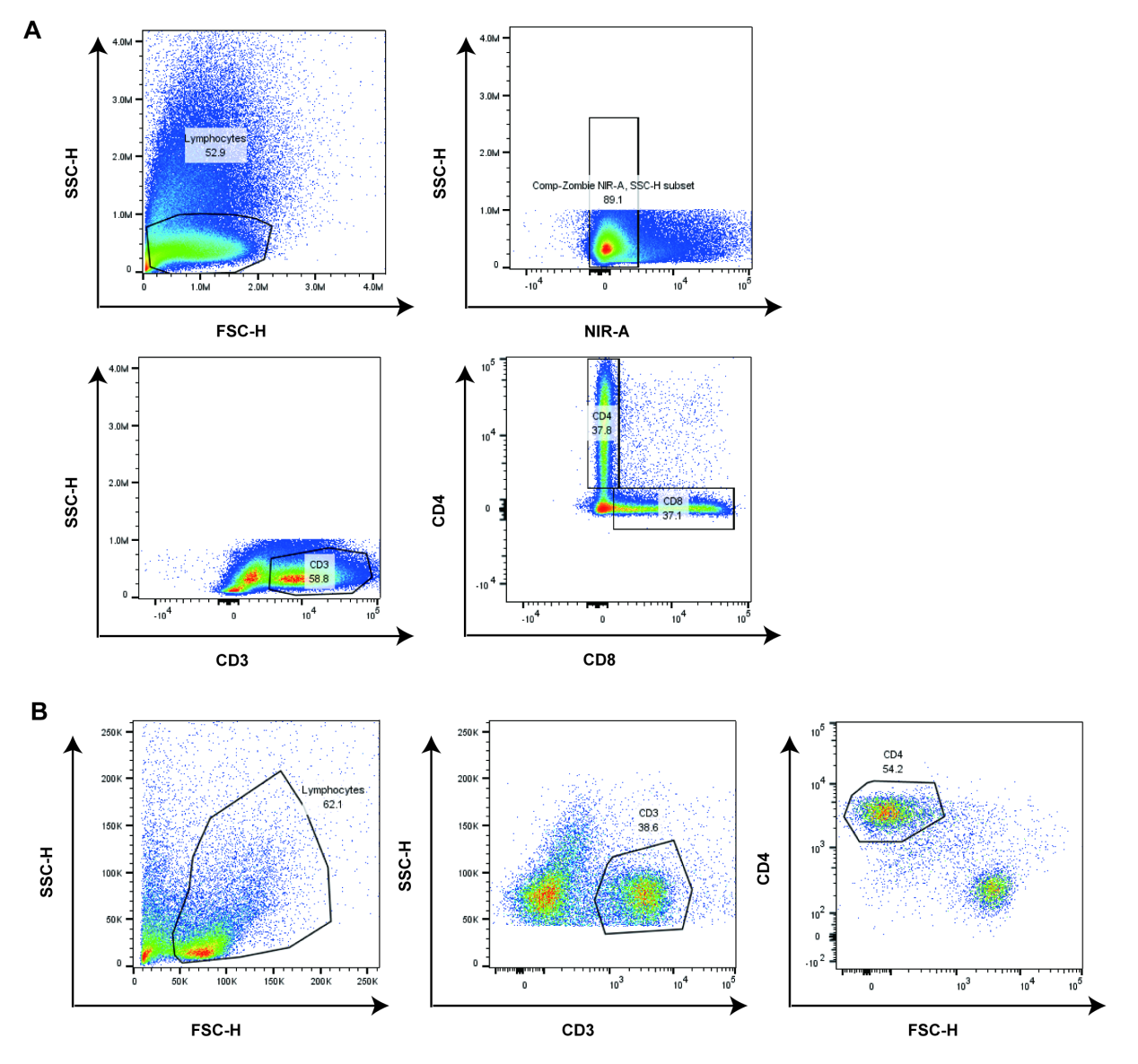
**

**Gating strategy for CD4^+^ T cells.**

Flow Gate strategy for CD4^+^ T cells in the patients with TLE (A)and TLE rats (B).

**Fig. S2.**

**
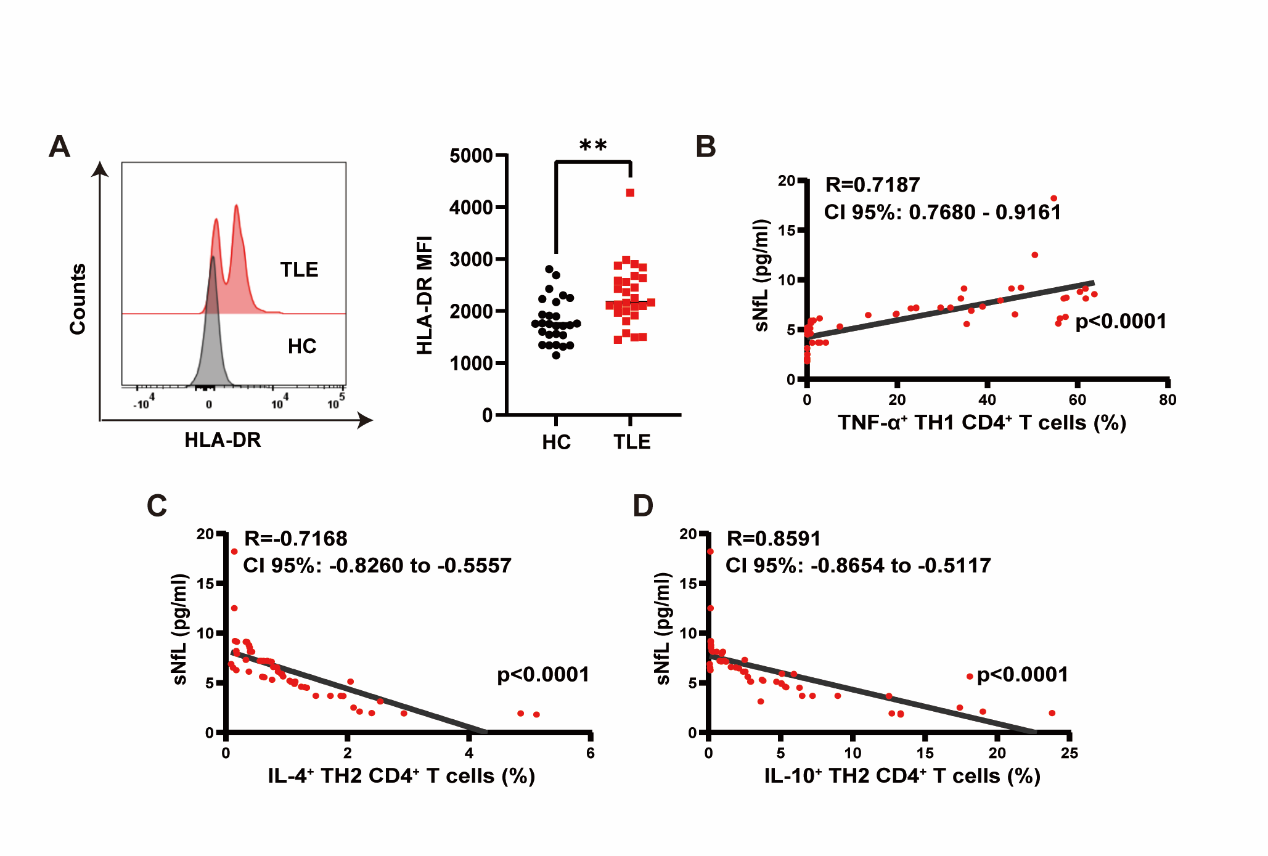
**

**CD4^+^ T cells are activated and their Th1/Th2 subtypes correlate with neuronal damage markers sNFL in the peripheral blood of patients with temporal lobe epilepsy (TLE).**

(A) Representative mean fluorescence intensity (MFI) curves and HLA-DR expression on CD4^+^ T cells from patients with TLE compared to healthy controls. Correlation (Pearson) of sNfL levels with the proportion of TNF-α^+^ Th1 CD4^+^ T cells(B), IL-4^+^ Th2 CD4^+^ T cells(C) and IL-10^+^ Th2 C CD4^+^ T cells (D). Data are presented as Mean ± standard deviation. *P<0.05, **P<0.01, ***P<0.001,****P<0.0001.

**Fig. S3.**

**
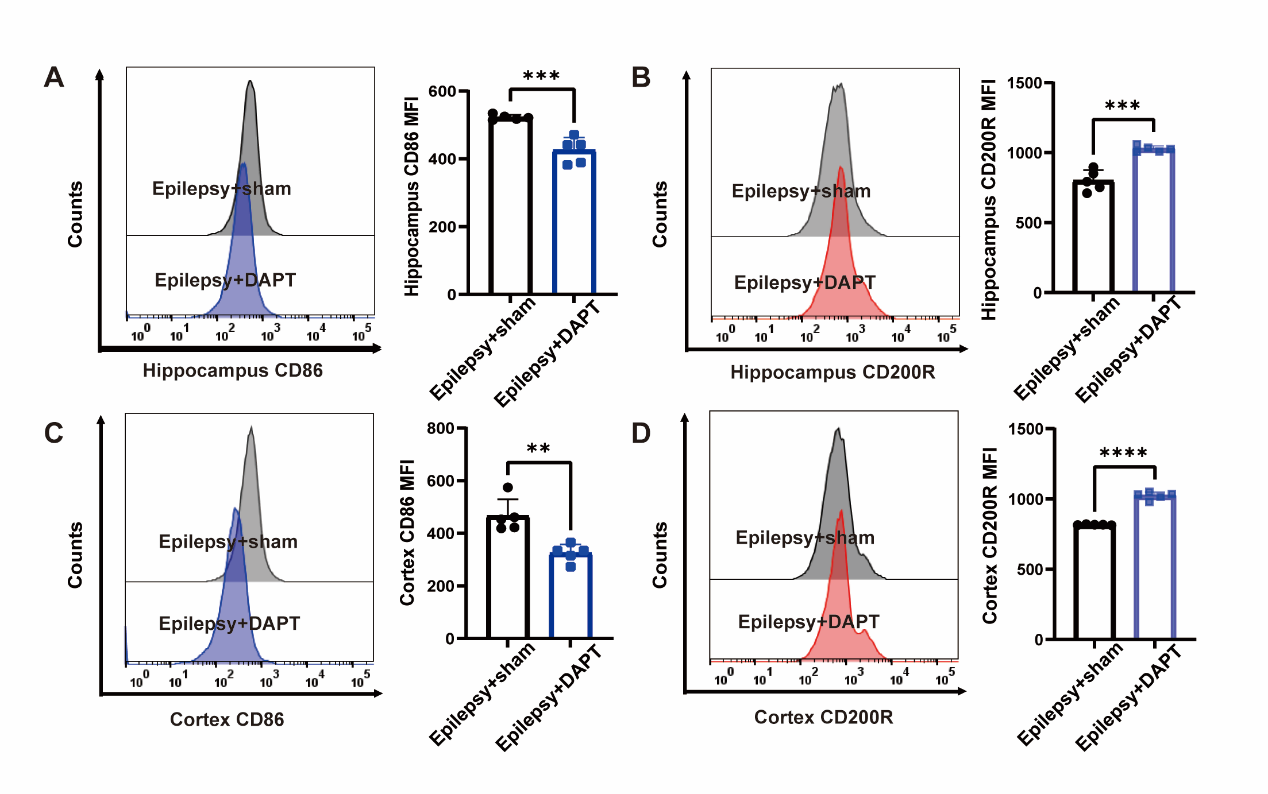
**

**DAPT modulates the microglia state dynamics in the CNS of rats with TLE.**

Representative MFI curves and expression of CD32(A) and CD200R(B) in the hippocampus of TLE rats compared to TLE rats treated with DAPT. Representative MFI curves and expression of CD32(C) and CD200R(D) in the cortex of the two groups. n=5 per group. Data are presented as Mean ± standard deviation. *P<0.05, **P<0.01, ***P<0.001, ****P<0.0001.
